# CSTF2 Promotes Hepatocarcinogenesis and Hepatocellular Carcinoma Progression *via* Aerobic Glycolysis

**DOI:** 10.3389/fonc.2022.897804

**Published:** 2022-07-08

**Authors:** Zhimin Chen, Weijie Hao, Jingzhi Tang, Wei-Qiang Gao, Huiming Xu

**Affiliations:** ^1^ State Key Laboratory of Oncogenes and Related Genes, Renji-Med X Clinical Stem Cell Research Center, Ren Ji Hospital, School of Medicine, Shanghai Jiao Tong University, Shanghai, China; ^2^ Department of Ultrasound, Fudan University Shanghai Cancer Center, Shanghai, China; ^3^ Med-X Research Institute and School of Biological Medical Engineering, Shanghai Jiao Tong University, Shanghai, China

**Keywords:** CSTF2, hepatocellular carcinoma, prognostic biomarker, aerobic glycolysis

## Abstract

**Background:**

The shortening of 3’ untranslated regions (3’UTRs) of messenger RNAs(mRNAs) by alternative polyadenylation (APA) is an important mechanism for oncogene activation. Cleavage stimulation factor 2 (CSTF2), an important regulator of APA, has been reported to have a tumorigenic function in urothelial carcinoma of the bladder and lung cancers. However, the tumor-promoting role of CSTF2 in hepatocellular carcinoma (HCC) and its underlying molecular mechanism remains unclear.

**Methods:**

Multiple databases were used to analyze the expression level and prognostic value of CSTF2 in HCC. Function enrichment analysis was used to investigate the molecular mechanism of CSTF2 for the occurrence and development of HCC. The biological function in HCC cell lines *in vitro* was determined by CCK8, colony formation, Transwell migration, and invasion assay. Moreover, the tumorigenic function of CSTF2 *in vivo* was measured by a subcutaneous tumor formation or injecting four plasmids into a mouse tail vein within 5–7 s in an immunocompetent HCC mouse model. In addition, aerobic glycolysis in HCC cells was determined by measuring the extracellular acid rate (ECAR) and extracellular glucose and lactate levels.

**Results:**

Bioinformatics analysis revealed that CSTF2 was overexpressed in HCC tissues. The high expression of CSTF2 was correlated with a poor prognosis and high histological grades. CSTF2 knockout inhibited the proliferation, migration, and invasion of HCC cells. In addition, CSTF2 knockout HCC cells failed to form tumors by a subcutaneous graft experiment. Furthermore, endogenous CSTF2 knockout attenuated hepatocarcinogenesis in an immunocompetent HCC mouse model. Function enrichment analysis suggested that the high expression of CSTF2 was associated with enhanced glycolysis. Moreover, we found that CSTF2 knockout reduced the level of the short 3’ UTR isoform of hexokinase 2 and increased its level of long 3’UTR. Furthermore, CSTF2 knockout inhibited ECAR levels, glucose uptake, and lactate production.

**Conclusion:**

Our results indicated that CSTF2 is highly expressed in HCC and is correlated with a poor prognosis and high histological grade. The knockout of CSTF2 inhibits the tumorigenesis and procession of HCC both *in vitro* and *in vivo*. Moreover, CSTF2 is associated with enhanced glycolysis. Therefore, this study suggests that CSTF2 might be a new prognostic biomarker and therapeutic target for HCC.

## Introduction

Liver cancer is one of the most common cancers in the world (1). Hepatocellular carcinoma (HCC) is the predominant histopathologic form of liver cancer, accounting for approximately 75% of total cases (2). Despite the advancement of the combination treatment of surgical resection and chemotherapy, recurrence and metastasis are still the main drawbacks of the curative treatment of HCC (3–5). Targeted therapy based on the dysregulation of gene expression and their associated molecular pathways is a novel and promising treatment strategy in advanced or metastatic HCC (6, 7). Thus, understanding the underlying molecular mechanisms of HCC is essential to develop novel treatment strategies.

It is well known that oncogene activation plays a crucial role in the pathogenesis of malignant tumors. Recently, the shortening of the 3’ untranslated regions (3’UTRs) of mRNAs by alternative polyadenylation (APA) is reported as an important mechanism for the upregulation and activation of oncogenes (8). As 3’UTRs contain regulatory sequence elements that are recognized by miRNAs and RNA-binding proteins, mRNA isoforms subjected to APA with different 3’UTRs lengths differ in their stability, translatability, and sublocation in the cells (9). In human cancers, the 3’UTR shortening of some oncogenes causes the upregulation of oncogene expression through the cis-acting mechanism and escape of microRNA-mediated inhibition (8, 10). In addition, the 3’UTR shortening of tumor-suppressor genes leads to their inactivation through a trans-acting mechanism (11). It is reported that the mRNAs of genes with shorter 3’UTR in tumors tend to be more aggressive and cause a poor prognosis (12). Up to now, it remains to be determined as to what extent APA occurs in HCC patients and what is the molecular mechanism of 3’UTR selection by APA in HCC.

The APA of pre-mRNA is carried out by a machinery composed of four protein complexes that recognize the conserved sequence around the cleavage site of 3’UTR (13). Among these complexes, the cleavage stimulation factor (CSTF) complex is related to the rapid initiation of the 3’-end processing of pre-mRNA, consisting of three different polypeptide subunits: CSTF1, CSTF2, and CSTF3 (14). CSTF2 (CstF64) is an important global regulator of APA. In the presence of limited CSTF2, the distal and stronger polyA signal sites (PASs) are preferred, and proximal PASs are less efficiently used. However, when CSTF2 is abundant, it promotes the selection of proximal and weaker PASs, which prevents the transcription and use of distal and stronger PASs (9, 15, 16). A recent study showed that high level of CSTF2 induces the shortening of RAC1 3’UTR, which is beneficial to the tumorigenesis of the urothelial carcinoma of the bladder. Furthermore, the high expression of CSTF2 with the short-3’UTR isoform of RAC1 is associated with a more invasive and poorer prognosis (17). In addition, the overexpression of CSTF2 appears to promote the growth and invasion of lung cancer cells (18). However, the tumor-promoting function and molecular mechanism of CSTF2 in HCC remain unclear.

In this study, we first comprehensively analyzed the expression of CSTF2 in HCC using multiple HCC databases and then investigated the association between the expression of CSTF2 and a prognosis using multiple bioinformatics analysis tools. Next, we performed *in vitro* and *in vivo* experiments to explore the function and molecular mechanism of CSTF2 for the tumorigenesis and development of HCC.

## Materials and Methods

### Data Collection

The gene expression data and corresponding clinical information were obtained from The Cancer Genome Atlas (TCGA, https://portal.gdc.cancer.gov/), Gene Expression Omnibus (GEO) (https://www.ncbi.nlm.nih.gov/geo/), and the International Cancer Genome Consortium (ICGC) portal (https://dcc.icgc.org/projects/LIRI-JP). In addition, the paired tumor and non-tumor liver tissues of 159 patients with liver cancer were the cohorts of Chinese patients with hepatitis B virus (HBV) infection (CHCC-HBV) (19) who underwent primary radical resection in Zhongshan Hospital (Fudan University, Shanghai, China) from 2010 to 2014. The data details are shown in **Supplementary Table S1**.

### Functional Enrichment Analysis

CSTF2-related genes were obtained by Spearman correlation analysis with correlation coefficients greater than or equal to 0.5. Next, the “clusterProfiler” R software package (20) was used for Gene Ontology (GO) and Kyoto Encyclopedia of Genes and Genomes (KEGG) pathway analyses, and ρ < 0.05 indicated that the difference was statistically significant. In addition, the Cancer Genome Atlas-Liver hepatocellular carcinoma (TCGA-LIHC) tumor samples were divided into high- and low-expression groups according to the median value of CSTF2, and gene set enrichment analysis (GSEA) was performed using the GSEA software package (version 4.0.3). “h. all. v6.2. entrez. gmt” and “c2. cp. kegg. v6.2. symbols. gmt” were used as reference gene sets.

### Cell Culture

HEK 293T cells and human hepatoma cell lines Huh7, MHCC-97H, and Hep3B were purchased from the Chinese Academy of Sciences Cell Bank (Shanghai, China). The cells were cultured in Dulbecco’s modified Eagle's medium (DMEM) (Gibco, USA) supplemented with 10% fetal bovine serum (FBS) (Sigma-Aldrich, MO,USA) and 1% penicillin/streptomycin (P/S, Gibco, USA) at 37°C with 5% CO_2_.

### Establishment of Stable CSTF2 Knockout and Overexpression of Hepatocellular Carcinoma Cell Lines

We constructed an sgRNA-targeting CSTF2 plasmid with the Lenti-CRISPR-V2 (Addgene #52,961) vector based on the CRIPR-Cas9 strategy using the following primers to construct a CSTF2-sgRNA plasmid: sgRNA-F: CACCGACAGGAAAGCCAAAGGTTA, sgRNA-R: AAACTAACCCTTTGGCTTTCCTGTC. The lenti-CRISPR-V2 empty vector was used as the control of CSTF2-sgRNA. In addition, we constructed a CSTF2 overexpression plasmid (pLVX-CSTF2-FLAG-puro). The empty vector pLVX-FLAG-puro was used as control. Next, CSTF2-sgRNA or overexpression plasmids together with the packaging plasmid psPAX2 (Addgene #12,260) and envelope plasmid pMD2.G (Addgene #12,259) were transfected into HEK-293T cells to be packaged into lentivirus. The virus supernatant was collected after 48 or 72 h, mixed with 8 μg/ml polybrene, and directly transfected the HCC cells. After 24 h of infection, 5 μg/ml puromycin was added for cell screening to establish a stable knockout or overexpression cell lines for cell screening. Finally, the efficiency of CSTF2 sgRNA or overexpression-stabilizing cell lines was identified by Western blotting.

### Western Blotting

Western blotting was performed as previously described (21). Briefly, the cells were lysed using a RIPA lysis buffer (Thermo Fisher Scientific, Waltham, MA, USA) containing a protein inhibitor cocktail (Merck, Kenilworth, New Jersey, USA). The protein concentration was determined by the BCA kit (Thermo Fisher, Waltham, MA, USA). The polyvinylidene fluoride (PVDF) membranes were blocked with non-fat milk and incubated with the primary antibodies against β-actin mouse antibody (1:1,000, ABclonal, China) and CSTF2 rabbit pAbs (1:1,000, ABclonal, Wuhan, China) overnight at 4°C, washed with TBST and then incubated with the corresponding HRP-conjugated secondary antibodies (Thermo Fisher Science, USA) for 1 h at room temperature (RT) and washed with a Tris-buffered saline with Tween-20 (TBST) buffer. The BioRad VersaDoc 4000 imaging system was used for imaging, and the densitometric analysis of proteins was quantified by Quantity One software (BioRad, Berkeley, CA, USA).

### CCK-8 Assay and Colony Formation Assay

Cell proliferation was measured by a CCK-8 (Dojondo Kumamoto, Japan) kit. The cells were inoculated with 10% CCK-8 at 37°C at 0, 24, 48, and 72 h; the optical density was measured at 450 nm in a microplate reader (BioTek, Winooski,Vermont, USA). Experiments were repeated three times independently. To assess the ability of the colony formation of HCC cells, the cells were seeded in 6-well plates at a density of 1,000 cells per well and cultured for approximately 14 days and formed an obvious colony. Then, the cells were fixed with 4% paraformaldehyde (PFA) for 15 min, stained with 0.2% crystal violet (Solarbio, China) for 30 min, and photographed.

### Transwell Migration Assay

Cell migration was determined by Transwell chambers with an 8-μm pore size (Corning, Corning NY, USA). HCC cells (4×10^4^ cells) were seeded into the upper chamber of transwell in serum-free DMEM. DMEM medium containing 10% FBS was added into the lower chamber. After incubation for 48 h, the non-migrating cells on the upper chamber were carefully removed by cotton swabs. The migrated cells attached to the lower side of the membrane were fixed with 4% PFA for 15 min and stained with 0.2% crystal violet. Then, the cells were washed with PBS three times (5 min each time) and visualized using an inverted microscope (Leica, Germany) and counted.

### Transwell Invasion Assay

Cell invasion was evaluated by a Transwell chamber with an 8-μm pore size (Corning, New York, USA). Firstly, Matrigel (1:8 dilution) precoated the upper chamber of the Transwell at 37°C for 2 h. Then, HCC cells (4 ×10^4^) were seeded into the upper chamber of transwell in a serum-free DMEM, and the DMEM medium supplemented with 10% FBS was added in the lower chamber. After incubation at 37°C for 48 h, the non-invasive cells on the upper chamber of the filter were removed and stained with 0.2% crystal violet and finally visualized using an inverted microscope and counted in the six random regions to quantify the number of invasive cells.

### Xenografts

Four-week-old male BALB/c nude mice were purchased from Shanghai SLAC Co. Ltd. (Shanghai, China). The mice were randomly grouped into six mice for each group. Huh7 cells (1 × 10^6^) infected with lentivirus containing CSTF2-sgRNA or a vector were subcutaneously injected into the right back of each mouse. The mice were sacrificed after 28 days, and the tumors were isolated and photographed. All animals were approved by the Institutional Animal Care and Ethics Committee of Renji Hospital with a maximum allowable tumor volume of 2,000 mm^3^.

### Hydrodynamic Injection Model

First, we constructed mouse CSTF2-sgRNA plasmids based on CRISPR-Cas9 methods using the following primers to construct CSTF2-sgRNA plasmids containing two sgRNAs plasmids: sgRNA-F1: CACCGCAGCGGGAGAGCACTTCGAG, sgRNA-R1: AAACTGCGGAACTTGAATGGGCGT, sgRNA-F2: ACGCCCATTCAAGTTCCGCA, sgRNA-R2: CTCGAAGTGCTCTCCCGCTGC. Then we confirmed the CSTF2-sgRNA plasmid by sequencing. Six-week-old female C57BL/6 mice were randomly grouped into six mice for each group. Approximately 2 ml of the plasmid mixture in 0.9% sodium chloride solution were prepared for the tail vein injection of each mouse. The plasmid mixture contained 13 μg pT3-EF1A-MYC-IRES-luc (Addgene 129775) (22), 13 μg pX330-p53 (Addgene 59910) (23), and 6.5 μg CMV-SB13 transposase. In the experimental group, 13 μg CSTF2-sgRNA (sgCSTF2) was added, while in the control group, a 13-μg empty vector lentiCRISPR v2 was mixed. Then, the plasmid mixture in sodium chloride solution was injected into the caudal vein of mouse within 5–7 s. Mice were monitored by abdominal palpation and euthanized when they had a high burden of liver tumors (i.e., large abdominal masses).

### Immunohistochemistry

The liver cancer tissues of mice were fixed with 4% PFA and embedded in paraffin. Approximately 5-μm thick slices were prepared for immunohistochemical staining. The sections were blocked with 10% normal donkey serum in PBST (Gibco, USA) for 1 h and incubated with primary antibodies against CSTF2 (1:200, Proteintech, China) or Ki67 (1:1000, Abcam, Cambridge, United Kingdom, Rabbit) overnight at 4°C. After being washed with PBS, the sections were incubated with biotinylated anti-rabbit Immunoglobulin G (IgG) secondary antibodies for 1 h at RT (1:200, Vector, Peterborough, United Kingdom). Then, the sections were visualized with a Vectastain ABC kit (Vector) at RT for 30 min and developed with 3,3’-diaminobenzidine (DAB) (Genetech, Shanghai, China) at RT. The images were visualized using a microscope. Tissue array chips were purchased from Servicebio (ZL-LivHCC961, Shanghai, China), and included HCC tissues (n = 48) and adjacent normal liver tissues (n = 48). Immunohistochemistry was executed on a tissue array chip using CSTF2 (1:200, Proteintech, China).

### Quantitative Real-Time PCR

Total RNA was extracted using TRIzol reagent (Life Technologies, #15596018) and reverse transcribed into cDNA using the PrimeScript RT reagent kit (Takara, Shiga, Japan). Quantitative real-time PCR (RT-PCR) were performed using Roche Light Cycler 480 detection system by SYBR Green PCR Master Mix kit (Toyobo, Osaka, Japan) and normalized by the expression of β-actin. The relative amount of each gene was quantified by the 2^−ΔΔCt^ method. All quantitative RT-PCR experiments were performed at least three independent experiments for culture cells. The information of the primers was listed in **Supplemental Table 2**.

### RNA 3’RACE

The total RNA of the cells was extracted using the TRIzol reagent (Thermo Fisher Scientific, Waltham, MA, USA), and the 3’end of cDNA was obtained by 3’RACE PCR using the 3’-RACE kit (Sangon Biotech, Shanghai, China). The semi-nest PCR approach was adopted to improve the accuracy of PCR, in which primers F1 and the 3’RACE outer primer were used in first-round PCR; primers F2 and the 3’RACE inner primer were used in the second-round PCR. The sequences of gene primers are listed below: ρ

hexokinase 2 (HK2) F1 (5’-3’): CCGGGGACGAGCTCTTTGACCACATT

HK2 F2 (5’-3’): GCTTTCACTCAACCCCGGCAAGCAGAG

### Seahorse Analyses

The extracellular acidification rate (ECAR) was measured by the Seahorse XF96 Analyzer (Seahorse Bioscience/Agilent Technologies, North Billerica, MA, USA). In short, 1×10^4^ HCC cells were inoculated in a 96-well plate overnight at 37°C for adhesion. Before detection, the medium was replaced by a detection medium and performed an ECAR experiment with the EACR kit (Seahorse Bioscience, Agilent) according to the manufacturer’s protocol.

### Glucose and Lactate Measurement

To test glucose uptake and lactate production, we cultured HCC cells up to 80% confluency. Then, the cells were changed to a fresh medium and cultured for 24 h, and the supernatant was collected for detection. Next, the concentration of glucose and lactate was measured by a glucose and lactate kit (Cedex Bio) using a Cedex Bio biochemical analyzer (Roche Diagnostics, Indianapolis, IN, USA), and normalization was performed according to the total protein levels in each group.

### Statistical Analysis

A statistical analysis of bioinformatic analysis was performed by R software (version 4.0.3). The experimental data are presented as the mean ± SEM of at least three independent experiments, and the statistical analysis was assessed by SPSS software 22.0. Statistical significance was evaluated using Student’s t-test to compare two groups, and the chi-square test was used for the comparisons of categorical variables. Spearman correlation analysis was used to determine the relationship between CSTF2 and other genes. Kaplan–Meier survival analysis was used to analyze the effect of CSTF2 on OS and relapse-free survival (RFS) in patients with liver cancer. The log-rank test was used to determine the statistical significance. ρ < 0.05 indicated statistical significance. * ρ < 0.05, ** ρ < 0.01, *** ρ < 0.001, **** ρ < 0.0001.

## Results

### CSTF2 Is Upregulated in Hepatocellular Carcinoma

**Figure 1:**
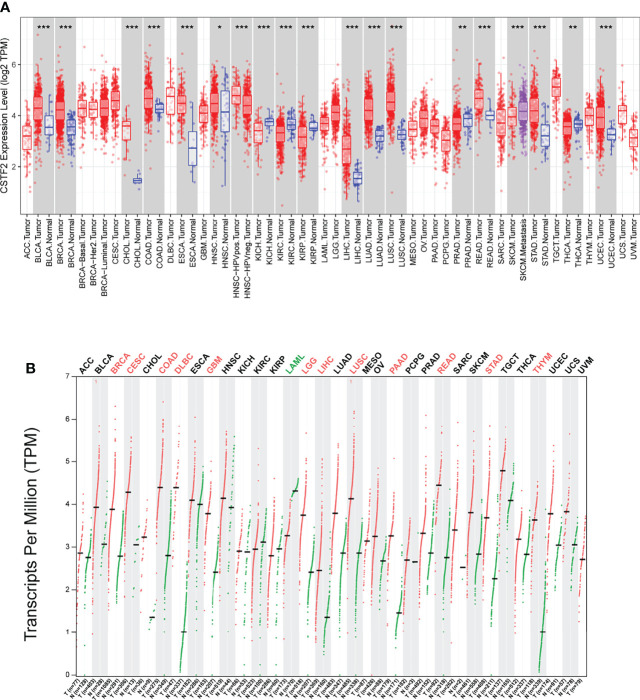
The expression levels of CSTF2 in human cancers. **(A)** The expression levels of CSTF2 in different tumor tissues compared to normal tissues in The Cancer Genome Atlas (TCGA) database. **(B)** The expression of CSTF2 in tumor tissues and matched TCGA normal in TCGA database and GTEx data by GEPIA analysis. * ρ < 0.05, ** ρ < 0.01, *** ρ < 0.001 compared to normal tissues.

**Figure 2:**
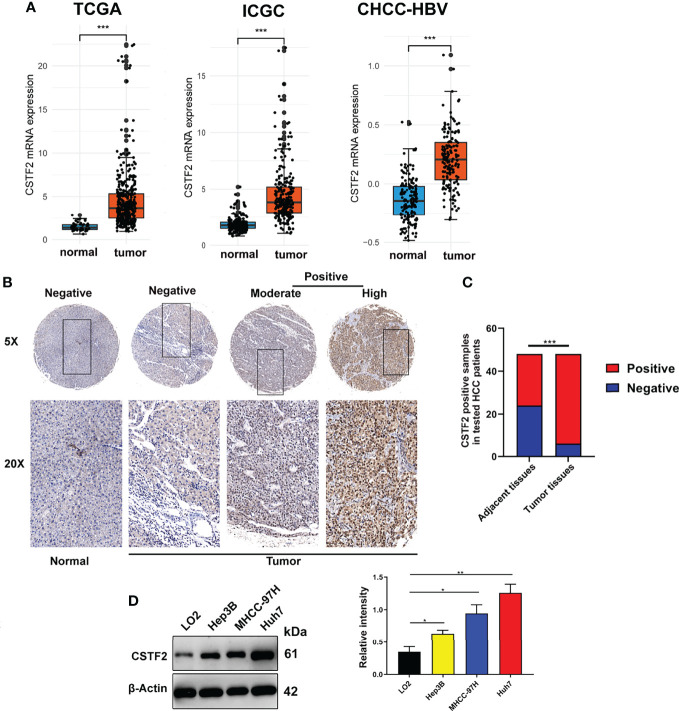
CSTF2 is highly expressed in HCC. **(A)** Analysis of CSTF2 mRNA expression levels in TCGA, International Cancer Genome Consortium (ICGC), and Chinese patients with hepatitis B virus (CHCC-HBV) databases **(B)** Representative images of the immunohistochemistry staining of CSTF2 in hepatocellular carcinoma **(HCC)** and adjacent non-tumor tissues from different HCC patients (tumor indicates HCC tissue; normal indicates adjacent normal tissue). **(C)** Quantitative analysis of sample numbers in different CSTF2 expression levels in 48 pairs of HCC tissues. **(D)** Western blot analysis of CSTF2 protein levels in non-tumorigenic LO2 liver cells, hepatocellular carcinoma cell lines Huh7, MHCC-97H, and Hep3B (left panel) and qualification of the relative intensity of CSTF2 protein levels (right panel). The levels of β-actin were used as an internal control. ** ρ < 0.01, *** ρ < 0.001.

To explore the tumorigenic function of CSTF2 in various tumors, we analyzed various databases to identify the expression level of CSTF2 in human tumors. We found that CSTF2 was highly expressed in plenty of cancers by analyzing the Tumor IMmune Estimation Resource (TIMER) database (24) (**Figure 1A**). Furthermore, CSTF2 is highly expressed in multiple human tumors and was also verified on TCGA and GTEx data by Gene Expression Profiling Interactive Analysis (GEPIA) (25) (**Figure 1B**). Subsequently, we confirmed the high expression of CSTF2 in HCC using other HCC databases from TCGA-LIHC, ICGC-JP, CHCC-HBV, and GEO (GSE36376, GSE112790, GSE14520, GSE76427, GSE25097, GSE124535) (**Figure 2A** and **Supplementary Figure S1**). Furthermore, the tissue microarray analysis of 48 pairs of HCC tissues also showed that CSTF2 was more strongly expressed in tumor tissues than in adjacent non-tumor tissues (**Figures 2B, 2C**, **Supplementary Figure S2**). In addition, we determined the expression levels of CSTF2 in HCC cell lines by Western blot and found that CSTF2 was expressed at a higher level in Huh7 and MHCC-97H, Hep3B cells than control, non-tumorigenic LO2 liver cells (**Figure 2D**). Collectively, the above data indicate that CSTF2 is upregulated in human HCC.

### High Expression of CSTF2 Is Associated With Patients’ Advanced Clinical Stages and a Poor Prognosis and Relapse in Hepatocellular Carcinoma

**Figure 3:**
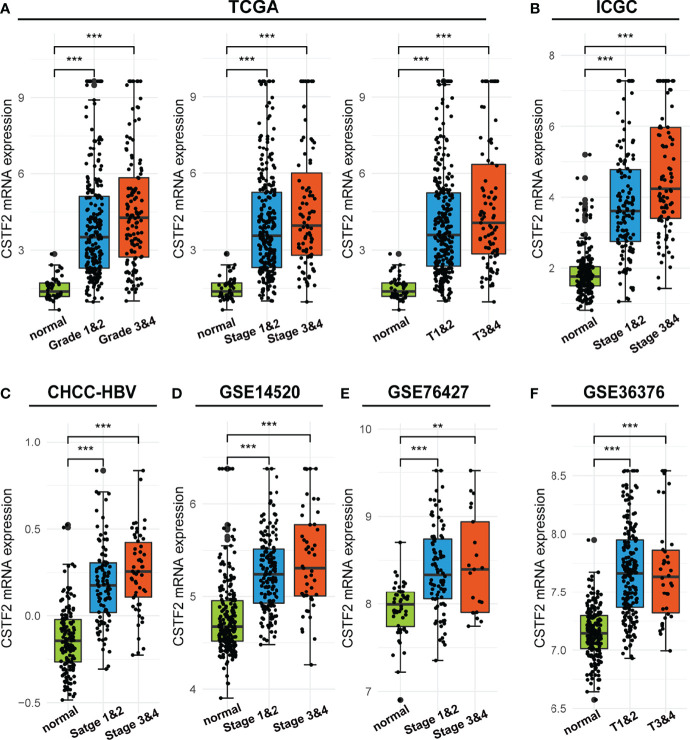
The mRNA levels of CSTF2 were correlated with the advanced clinical stage in HCC in different databases. **(A-F)** The relationship between CSTF2 expression levels with a histological grade, a clinical stage in TCGA databases, ICGC databases, CHCC-HBV databases, GSE14520 databases, and GSE76427 databases and in GSE36376 databases. ** ρ < 0.01, *** p < 0.001 compared to normal.

**Figure 4:**
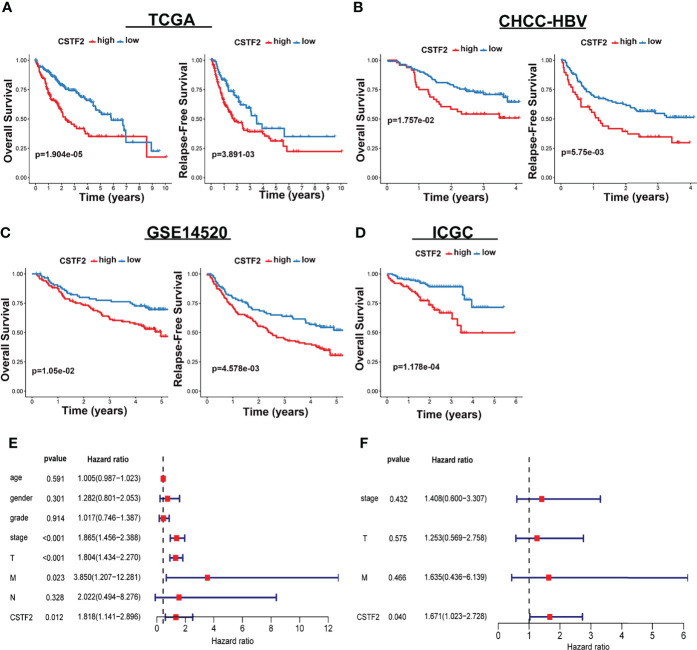
CSTF2 higher expression is associated with poor prognosis in HCC. **(A-D)** Overall survival and relapse-free survival in TCGA-LIHC, CHCC-HBV, GSE14520, and ICGC databases **(E)** Univariate and **(F)** multivariate Cox analyses of CSTF2 expression at different TNM stages, ages, and genders.

We further analyzed the correlation between high CSTF2 expression and the clinical malignant grades of HCC using the HCC databases from TCGA-LIHC, ICGC-JP, CHCC-HBV, and GEO (GSE14520, GSE76427, GSE36376). **Figure 3** shows that the higher expression of CSTF2, the more advanced clinical stage for HCC patients (ρ < 0.05), suggesting that the expression of CSTF2 was related to the clinicopathological characteristics of HCC patients. In addition, Cox proportional hazard analysis using the TCGA database showed that HCC patients with higher CSTF2 expression had poor overall survival (OS) and relapse-free survival (RFS) (**Figure 4A**). We further confirmed the above conclusion with other liver cancer databases including CHCC-HBV, GSE14520, and ICGC databases (**Figures 4B–D**). Furthermore, multivariate Cox analysis demonstrated that CSTF2 expression was an independent prognostic factor for OS [hazard ratio (HR) = 1.671, 95% confidence interval (CI): 1.023–2.728, ρ = 0.040] (**Figures 4E–F**). Taken together, these results indicated that the higher expression of CSTF2 is related to the advanced clinical stage and a poor prognosis for HCC patients.

### Knockout of CSTF2 Inhibits Cell Proliferation, Migration, and Invasion of Hepatocellular Carcinoma Cell Lines

**Figure 5:**
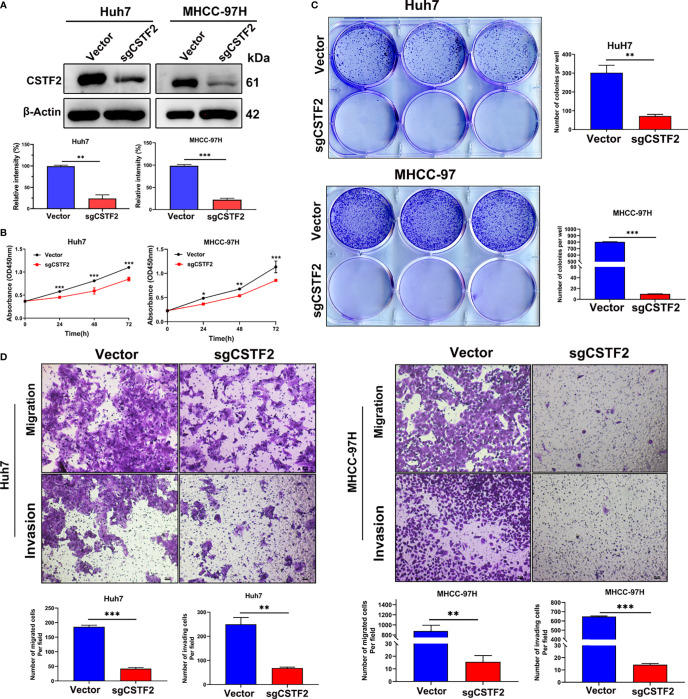
Knockout of CSTF2 inhibited the proliferation, migration, and invasion of HCC cells *in vitro*. **(A)** The stable CSTF2 knockout Huh7 and MHCC-97H cells were determined by Western blot (upper panel) and the qualification of the relative intensity of CSTF2 protein levels (bottom panel). The levels of β-actin were used as an internal control. **(B)** The cell proliferation ability of CSTF2 knockout Huh7 and MHCC-97H cells was measured by the CCK-8 assay. **(C)** Colony-forming assay of CSTF2 knockout Huh7 cells and MHCC-97H cells (left panel) and the qualification of the number of colonies in these cells (right panel). **(D)** The migration and invasion capacity were evaluated in CSTF2 knockout Huh7 cells and MHCC-97H cells by the Transwell assay (upper panel) and the qualification of migrated cells and invading cells per field (bottom panel). Scale bar, 50 μm. sgCSTF2 represents CSTF2-sgRNA. An empty vector was used as a control of CSTF2-sgRNA. * ρ < 0.05, ** p < 0.01, *** p < 0.001 compared to vector control.

To further determine the function of CSTF2 in HCC, we established stable CSTF2 knockout Huh7 and MHCC-97H cells by a CRISPR/Cas9 single-guide RNA (sgRNA) strategy. Western blotting showed that the expression levels of CSTF2 in CSTF2-sgRNA Huh7 and MHCC-97H cells were markedly lower than that in the vector control cells (**Figure 5A**). **Figure 5B** shows that knockout of CSTF2 significantly inhibited the cell proliferation in Huh7 and MHCC-97H cells by the CCk8 assay. Next, we studied the colony-forming capacity of HCC cells and found that CSTF2-sgRNA Huh7 cells and MHCC-97H cells formed far fewer colonies than the control vector group under low-density culture conditions (**Figure 5C**). Transwell assay showed that knockout of CSTF2 dramatically inhibited the migration and invasion of Huh7 cells and MHCC-97H cells (**Figure 5D**). These results together imply that the knockout of CSTF2 inhibits cellular proliferation, migration, and invasion of HCC cell lines.

### Overexpression of CSTF2 Promotes Cell Proliferation, Migration, and Invasion in Hepatocellular Carcinoma Cells

**Figure 6:**
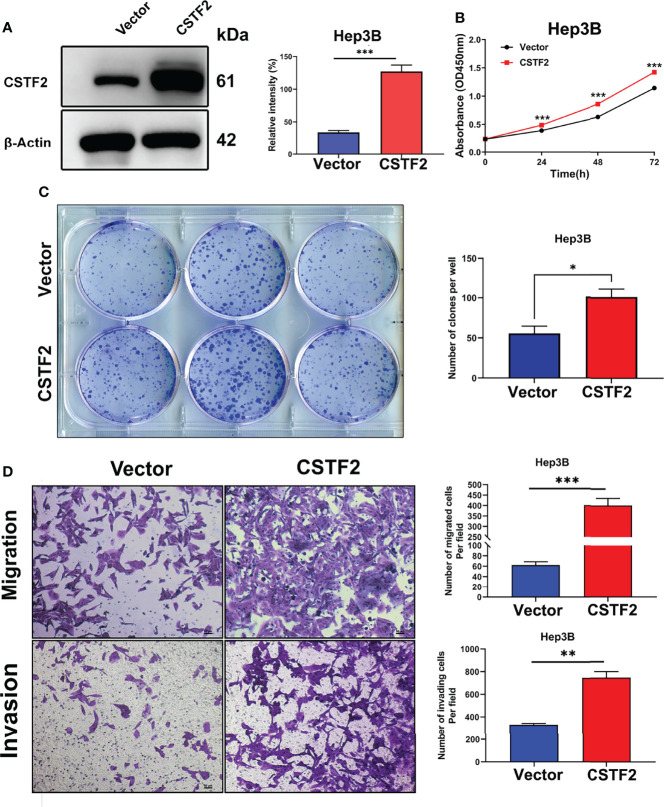
Overexpression of CSTF2 promoted the cell proliferation, migration, and invasion of HCC cells *in vitro*. **(A)** The stable overexpression of CSTF2 in Hep3B cells was determined by Western blot (left panel) and the qualification of the relative intensity of CSTF2 protein levels (right panel). **(B)** CCK-8 assay of the cell proliferation rate of the overexpression of CSTF2 Hep3B cells. **(C)** Colony-forming assay of the overexpression of CSTF2 Hep3B cells (left panel) and the qualification of the number of colonies formed (right panel). **(D)** The migration and invasion were measured in the overexpression of CSTF2 Hep3B cells by the Transwell assay (left panel) and the qualification of migrated cells and invading cells per field (right panel). CSTF2 represents the overexpression of CSTF2. An empty vector was used as control. Scale bar, 50 μm. * ρ < 0.05, ** ρ < 0.01, *** ρ < 0.001 compared to the vector.

To further verify the tumor-promoting function of CSTF2, we overexpressed CSTF2 in the HCC cell line Hep3B and confirmed that the expression level of CSTF2 was higher than that of vector control by Western blot (**Figure 6A**). **Figures 6B–6C** show that overexpression of CSTF2 evidently promoted the cell proliferation of Hep3B cells based on the CCk8 assay and colony-forming assay. Moreover, we confirmed the result in another HCC cell line sk-Hep1 cells (**Supplementary Figure S3**). In addition, the overexpression of CSTF2 also promoted cell migration and invasion in Hep3B cells by Transwell assays (**Figure 6D**). All in all, these results indicate that the overexpression of CSTF2 promotes cancer cell proliferation, migration, and invasion in HCC cells.

### Knockout of CSTF2 Inhibits Tumorigenesis and Processing of Hepatocellular Carcinoma *In Vivo*

**Figure 7:**
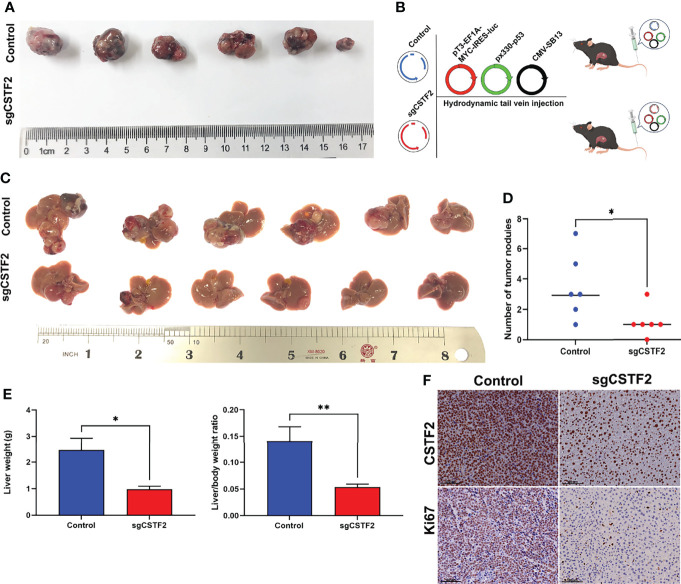
Knockout of CSTF2 inhibited the tumorigenesis and progression of HCC. **(A)** *In vivo* tumor formation of CSTF2 sgRNA Huh7 and vector control Huh7. Tumorigenesis experiments were performed by subcutaneously injecting Huh7 cells into BALB/c nude mice. **(B)** Strategy for evaluating the hepatocarcinogenesis function of CSTF2 in immunocompetent C57BL/6 mice. Schematic representation of the hydrodynamic tail vein injection HCC model. Plasmids pT3-EF1A-MYC-IRES-luc, px330-p53 (p53-sgRNA), and CMV-SB13 transposase were delivered together with either CSTF2 sgRNA (sgCSTF2) or the control vector for HCC induction. **(C)** Representative images of dissected livers in CSTF2 sgRNA and the vector control group. **(D)** The number of tumor nodules in mice injected with sgCSTF2 was significantly lower than that in the control group. **(E)** Qualification of the liver weight, and the ratio of liver weight to body weight of these tumor nodules in CSTF2 sgRNA or control groups. At least 6 mice were used in every group. **(F)** Immunohistochemical staining formed tumors in the liver tissues using CSTF2 and Ki67 antibodies. Scale bar, 100 μm. sgCSTF2 represents CSTF2-sgRNA. The empty vector of sgRNA was used as control. * ρ < 0.05, ** ρ < 0.01 compared to control.

Next, we extended our studies to an *in vivo* setting. We first performed subcutaneous tumor formation experiments by injecting 1×10^6^ cells into the right back of BALB/c nude mice in the vector control group and CSTF2 sgRNA group, respectively. Approximately 28 days later, these mice were sacrificed, and the tumors were removed and photographed. **Figure 7A** shows that the knockout of CSTF2 in Huh7 cells completely inhibited tumor formation, while there were bigger tumors formed in the control group, indicating that the knockout of CSTF2 could completely inhibit the tumor formation and growth of human HCC cells in BALB/c nude mice. To further confirm the inhibitory effect of CSTF2 in HCC, we performed the endogenous CSTF2 knockout by mouse CSTF2 sgRNA in an immunocompetent HCC mouse model by the hydrodynamic tail vein injection (HTVI) delivery method according to a previously reported protocol (22, 26). Briefly, we simultaneously injected four plasmids including pT3-EF1A-MYC-IRES-luc, CMV-SB13 (transposase), px330-p53 (p53 sgRNA), sgCSTF2 (CSTF2 sgRNA containing double CSTF2 sgRNAs), or empty vector control (Control) into the mouse tail vein within 5–7 s (**Figure 7B**). Thirty-five to forty days later, tumors would be formed in the liver tissue of mice. As shown in **Figures 7C–E**, the endogenous CSTF2 knockout significantly reduced the size, numbers of tumor nodules, and liver weight compared with the control group. Moreover, the immunohistochemical staining of liver tumor nodules confirmed that the expression of CSTF2 in the CSTF2 sgRNA group was much lower than that in the control group. In addition, the expression of Ki67 in the CSTF2 sgRNA group was significantly lower than that in the control group (**Figure 7F**). Taken together, the above results demonstrate that CSTF2 might play a crucial role in the tumorigenesis and progression of HCC.

### Biological Functions and Enrichment Pathways Related to CSTF2

**Figure 8:**
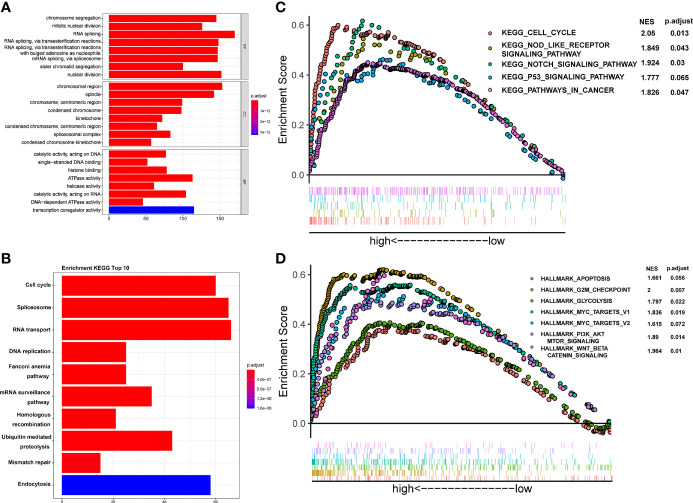
Functional enrichment analysis of CSTF2 in the TCGA cohort. **(A)** GO classification analysis of gene changes in the biological process. **(B)** KEGG pathway enrichment analysis of upregulated pathways **(C, D)** GSEA analysis of activated pathways and hallmarks of CSTF2 related in HCC based on the TCGA database.

To investigate the potential molecular mechanism of CSTF2 responsible for the tumorigenesis and progression of HCC, GO, KEGG, and GSEA enrichment analyses were performed based on the TCGA-LIHC database. GO and KEGG enrichment analyses showed that CSTF2 might be related to RNA splicing, DNA replication, chromosome segregation, and cell cycle–related biological processes involving cell proliferation (**Figures 8A–B**). Moreover, GSEA analysis indicated that CSTF2 high expression was also associated with cancer-related pathways, such as Myelocytomatosis (MYC) targets, P53 signaling, the Nod-like receptor signaling pathway, glycolysis, Wnt/beta-catenin, and PI3K/Akt/mTOR signaling, which play an important role in promoting tumorigenesis and the progression of HCC (27–31) (**Figures 8C–D**). Altogether, it appears that the high expression of CSTF2 activates many cancer-related pathways and cell-cycle and cell-proliferation-related pathways, contributing to the growth and development of HCC.

### High Expression of CSTF2 Enhances Aerobic Glycolysis in Hepatocellular Carcinoma Mainly by Enhancing HK2 3’UTR Short Isoform

**Figure 9:**
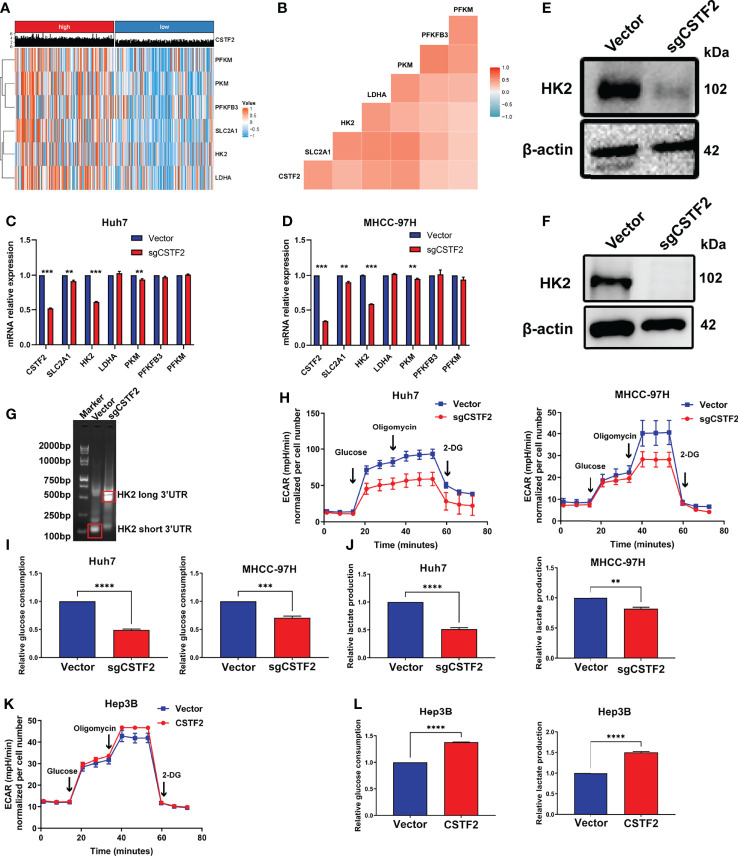
High expression of CSTF2 enhances aerobic glycolysis in HCC. **(A)** Heat map of the cluster analysis of the relationship between the CSTF2 expression and glycolysis-related enzymes and glucose transporters. **(B)** The correlation analysis of CSTF2 expression and glycolysis-related enzymes and glucose transporters. **(C-D)** The relative expression levels of CSTF2, SLC2A1, HK2, LDHA, PKM, PFKFB3, and PFKM detected by quantitative RT-PCR in Huh7 cells and MHCC-97H cells. **(E–F)** The protein levels of HK2 in Huh7 cells and MHCC-97H cells determined by Western blot; the levels of β-actin were used as an internal control. **(G)** 3’ RACE HK2 fragments were amplified in Huh7 cells and detected by agarose gel electrophoresis. **(H–J)** The glycolytic function of HCC cells was measured by the extracellular acidification rate (EACR), relative glucose uptake, and lactate production in CSTF2 knockout Huh7 and MHCC-97H cells. (K-L) The glycolytic function of HCC cells was measured by EACR, the relative glucose uptake, and lactate production in CSTF2-overexpression Hep3B cells. sgCSTF2 represents CSTF2 sgRNA, CSTF2 represents CSTF2 overexpression, and the vector was used as control. Data were presented as the mean ± SEM. ** ρ < 0.01, *** ρ < 0.001 compared to the vector.

A growing body of evidence indicates that aerobic glycolysis or the Warburg effect plays an important role in the proliferation, invasion, and metastasis of HCC (31) and is also involved in the mechanism of sorafenib resistance in HCC patients (32). Here, our GSEA analysis showed that the high expression of CSTF2 is associated with the glycolysis pathway (**Figure 8D**). Considering that the high expression of CSTF2 is also associated with the activated PI3K/Akt/mTOR pathway and MYC target signals that promote glycolysis (31, 33, 34), we further analyzed the relationship between CSTF2 expression levels and glycolysis. According to the median value of CSTF2 expression, we divided the TCGA-LIHC data into two groups: high and low expression. The cluster heat map of glycolysis-related key enzymes and transporters including hexokinase 2 (HK2), phosphofructokinase 1 isoform found in muscle (PFKM), fructose-2,6-bisphosphatase 3 (PFKFB3), pyruvate kinase 2 (PKM2), L-lactate dehydrogenase A(LDHA), and glucose transporter member 1 (GLUT-1, also named SLC2A1) were highly expressed in the CSTF2 high-expression group (**Figure 9A**). Moreover, the expression of these genes was positively correlated with the expression of CSTF2 (**Figure 9B**). Next, we detected the expression of the above glycolysis-rated enzymes and transporters by quantitative RT-PCR and found that the levels of HK2, PKM, and SLC2A1 in CSTF2-sgRNA Huh7 and MHCC-97H cells were lower than that in the vector control cells, especially HK2 (**Figures 9C, D**). We further detected the protein levels of HK2 in the cells by Western blot. As shown in **Figures 9E, F**, knockout CSTF2 dramatically decreased the protein levels of HK2. Next, we wondered if the knockout of CSTF2 could decrease the levels of the short 3’UTR isoform of HK2. Interestingly, **Figure 9G** shows that that the levels shorter-3'UTR isoform were significantly decreased in CSTF2-sgRNA Huh7 cells compared to that in the vector control cells, but the longer-3’UTR isoform was increased in CSTF2-sgRNA Huh7 cells. These results suggest that high levels of CSTF2 enhance HK2 3’UTR shorting and promote the glycolysis process.

To confirm the glycolysis, we measured the extracellular acid rate (ECAR) of HCC cells using the Seahorse metabolic analyzer. **Figures 9H–9J** show that knockout of CSTF2 reduced the glycolytic function in both Huh7 and MHCC-97H cells, evidenced by decreased ECAR, a marked reduction in glucose uptake, and extracellular lactate levels in CSTF2 sgRNA Huh7 and MHCC-97H cells. By contrast, the overexpression of CSTF2 led to a significant increase in ECAR, glucose uptake, and extracellular lactate levels in Hep3B cells (**Figures 9K, 9L**). All in all, the above results indicate that the high expression of CSTF2 enhances aerobic glycolysis in HCC.

## Discussion

In the current study, we demonstrated that CSTF2 is highly expressed in tumor tissues compared with that in normal tissues in a variety of malignant tumors based on the TCGA pan-cancer database. It is worth noting that CSTF2 is highly expressed in HCC tissues based on plenty of databases. Moreover, the HCC tissue microarray confirmed that the expression of CSTF2 in tumor tissues is much higher than that in adjacent non-tumor normal tissues from different HCC patients. More importantly, the high expression of CSTF2 is associated with advanced clinical stages and a poor clinical prognosis by univariate and multivariate Cox regression analyses. Therefore, the high expression of CSTF2 could be an independent prognostic biomarker for HCC patients.

The current study has investigated the tumorigenic function of CSTF2 not only *in vitro* but also *in vivo*. Firstly, the knockout of CSTF2 significantly inhibits the cell proliferation, migration, and invasion of HCC cells *in vitro*. Secondly, the overexpression of CSTF2 promotes the cell proliferation, migration, and invasion of HCC cells in cell cultures. Thirdly, Huh7 cells stably transfected with CSTF2-sgRNA are unable to form tumors by a subcutaneous graft experiment. Fourthly, an endogenous CSTF2 knockout distinctly reduces the carcinogenic ability in an immunocompetent HCC mouse model. Collectively, these results demonstrate clearly that CSTF2 plays an important role in the tumorigenesis and progression of HCC.

Of note, in the present study, we adopted an immunocompetent HCC mouse model by the hydrodynamic tail vein injection delivery method to measure the carcinogenic ability of CSTF2. In the model, by the overexpression of oncogenes (transposon-based vectors) and the deletion or mutation of tumor suppressor genes (using the CRISPR-Cas9 system), liver cancer models can be quickly established. Moreover, the model can replicate the interaction between tumor cells and the immune system, reproduce tumor development and immune response, and allow for interrogating the role of any genetic alteration in an antitumor immunity system (22).

In terms of the molecular mechanism of CSTF2 on HCC, GO and KEGG enrichment analyses revealed that the high expression of CSTF2 is related to the cell cycle and DNA replication, which contribute to tumor proliferation (27). The present GSEA analysis indicates that CSTF2 is involved in the Notch signaling pathway, P53 signaling pathway, glycolysis, PI3K/Akt/mTOR signaling pathway, and Wnt-β catenin signaling pathway, which is involved in the tumorigenesis and development of tumors (27–31). Of note, aerobic glycolysis, known as the Warburg effect, is the metabolic phenotype of cancer cells (35) and can meet the needs of the rapid growth of tumors (36). Previous studies have demonstrated that suppressing aerobic glycolysis can inhibit the growth of HCC (37, 38). In this regard, it is particularly important to note that the present work demonstrates the involvement of CSTF2 in glycolysis. The correlation analysis reveals that the high expression of CSTF2 is positively related to the transcription of glycolysis-related limiting enzymes and the key glucose transporter factor SLC2A1. In our study, we found that the expression of the rate-limiting enzyme HK2 in aerobic glycolysis was dramatically reduced during the knockout of CSTF2. Moreover, we found that the knockout of CSTF2 evidently reduced the levels of the shorter-3’UTR isoform of HK2 and increased the levels of the longer-3’UTR isoform of HK2. As the main regulator of 3’UTR shortening in many types of tumors, CSTF2 may induce the 3’UTR shortening of HK2 and make it escape the inhibition mediated by microRNA, leading to enhanced glycolysis. Therefore, the finding indicates that the high expression of CSTF2 could induce the HK2 3’UTR shortening of HK2 and promote the expression of HK2, resulting in tumorigenesis and progression in HCC. Supporting our finding, previous studies have demonstrated that HK2 is highly expressed in HCC and is directly related to the pathological stage and prognosis of patients (39). Finally, toward this direction, GSEA analysis showed that the high expression of CSTF2 is also associated with the activated PI3K/Akt/mTOR pathway and MYC target signals, which promote glycolysis by increasing the glucose uptake rate (40, 41) and upregulating the expression and activity of glycolytic enzymes and HIF-1a (31, 33, 34). However, how HK2 was regulated by CSTF2 needs more investigation in the future. Therefore, our results suggest that CSTF2 promotes the progression of HCC by enhancing the Warburg effect.

It is given that cancer stem cells (CSCs) prefer to take glycolysis for energy production (42), and the inhibition of glycolysis reduces the number of CSCs (43). Our GSEA analysis showed that the high expression of CSTF2 activates the Notch signaling and Wnt/β catenin, which play an important role in activating and maintaining the stemness of CSC in HCC (30). In addition, glycolysis contributes to cancer initiation, progression, chemoresistance, and relapse (42, 44). Therefore, our finding indicates that CSTF2 could play an important role in activating and maintaining the stemness of CSC in HCC, resulting in oncogenesis, processing, and relapse. However, whether CSTF2 influences the stemness of CSC in HCC remains to be determined in the future. Taken together, the above data indicate that the high expression of CSTF2 could enhance glycolysis, which might augment the stemness, promoting the tumorigenesis and progression of HCC.

## Conclusions

Our findings reveal that the high expression of CSTF2 is associated with a poor prognosis in HCC patients. Moreover, the high expression of CSTF2 promotes hepatocarcinogenesis and HCC progression by enhancing HK2 3’UTR shorting and aerobic glycolysis. Therefore, this study indicated that CSTF2 might be a novel clinical biomarker for prognosis and a molecular therapeutic target for HCC patients.

## Data Availability Statement

Publicly available datasets were analyzed in this study. This data can be found here: TCGA(https://portal.gdc.cancer.gov/),ICGC (https://dcc.icgc.org/projects/LIRI-JP), GEO (https://www.ncbi.nlm.nih.gov/geo/),MHCC-HBV(PMID: 31585088),All data generated and used in this study are available for anyone to utilize upon reasonable request.

## Ethics Statement

The animal study was reviewed and approved by Institutional Animal Care and Ethics Committee of Renji Hospital.

## Author Contributions

HX and W-QG conceived and designed the study. ZC and WH provided equal contributions to research design, data analysis, and article writing. JT helped to perform animal experiment. HX and W-QG reviewed the manuscript. The final manuscript has been read and approved by all authors.

## Funding

This work was funded by the funding of the National Natural Science Foundation of China (81630073 and 81872406) and 111 project (B21024).

## Conflict of Interest

The authors declare that the research was conducted in the absence of any commercial or financial relationships that could be construed as a potential conflict of interest.

The reviewer HJ declared a shared parent affiliation with the authors ZC, WH, W-QG, HX to the handling editor at the time of review.

## Publisher’s Note

All claims expressed in this article are solely those of the authors and do not necessarily represent those of their affiliated organizations, or those of the publisher, the editors and the reviewers. Any product that may be evaluated in this article, or claim that may be made by its manufacturer, is not guaranteed or endorsed by the publisher.
