# Supplementary material for: CSTF2 Promotes Hepatocarcinogenesis and Hepatocellular Carcinoma Progression via Aerobic Glycolysis
Source: Front Oncol. 2022 Jul 8;12:897804. doi: 10.3389/fonc.2022.897804 (PMC9304882; doi:10.3389/fonc.2022.897804)
Supplement: Supplementary file 1 [file DataSheet_1.docx]

Supplementary Material

| **Dataset** | **Normal (n)** | **Tumor (n)** |
| --- | --- | --- |
| LIHC ( TCGA) | 50 | 374 |
| LIRI-JP (ICGC) | 202 | 243 |
| CHCC-HBV | 159 | 159 |
| GSE36376 | 193 | 240 |
| GSE112790 | 15 | 183 |
| GSE14520 | 220 | 225 |
| GSE76427 | 52 | 115 |
| GSE25097 | 243 | 268 |
| GSE124535 | 35 | 35 |
| **Total** | 1169 | 1842 |

1. **Supplementary Table:**

Table S1. The detail of gene expression profiles of hepatocellular carcinoma.

**n presents the number of tumor patients.**

Table S2. Sequences for quantitative real-time PCR

| **Name** |  | **Primer Sequence 5’-3’** |
| --- | --- | --- |
| β-actin | Former | CACCATTGGCAATGAGCGGTTC |
|  | Reverse | AGGTCTTTGCGGATGTCCACGT |
| CSTF2 | Former | CAGGGTGGATCGTTCTCTAC |
|  | Reverse | AACAACAGGTCCAACCTCAGA |
| SLC2A1 | Former | GGCCAAGAGTGTGCTAAAGAA |
|  | Reverse | ACAGCGTTGATGCCAGACAG |
| HK2 | Former | CCTGAGGACATCATGCGAGG |
|  | Reverse | TGAGACCAGGAAACTCTCGTC |
| LDHA | Former | ATGGCAACTCTAAAGGATCAGC |
|  | Reverse | CCAACCCCAACAACTGTAATCT |
| PKM2 | Former | ATGTCGAAGCCCCATAGTGAA |
|  | Reverse | TGGGTGGTGAATCAATGTCCA |
| PFKFB3 | Former | ATTGCGGTTTTCGATGCCAC |
|  | Reverse | GCCACAACTGTAGGGTCGT |
| PFKM | Former | GGTGCCCGTGTCTTCTTTGT |
|  | Reverse | AAGCATCATCGAAACGCTCTC |

1. **Supplementary Figures:**


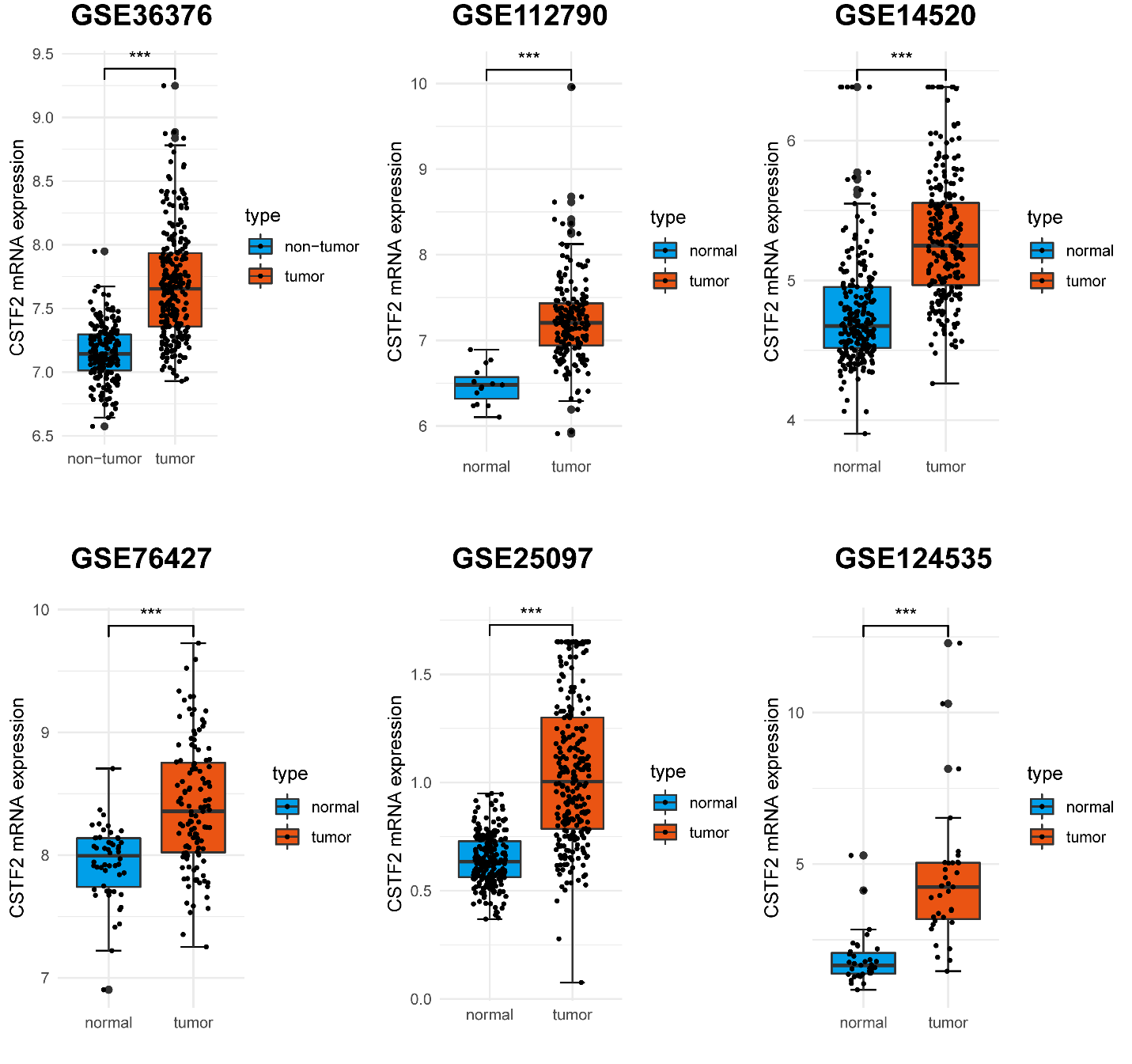


**Figure S1.** Relative Expression levels of CSTF2 in HCC from GEO database. ***$\text{ p}$ < 0.001.


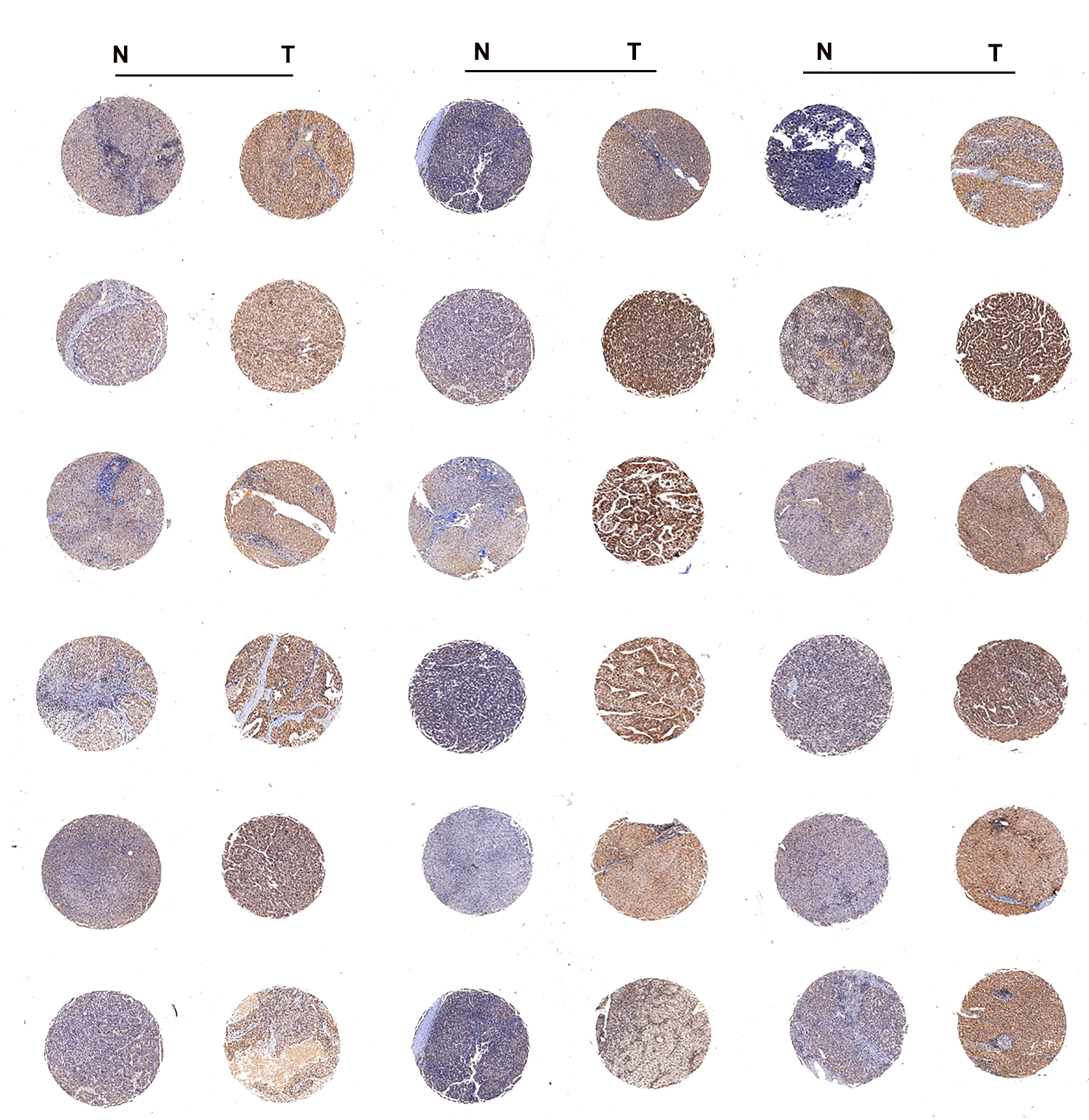


**Figure S2.** Representative immunohistochemistry images of CSTF2 expression in tissue microarrays of different HCC patients. T represents tumor tissues; N represents paired adjective non-tumor tissues.


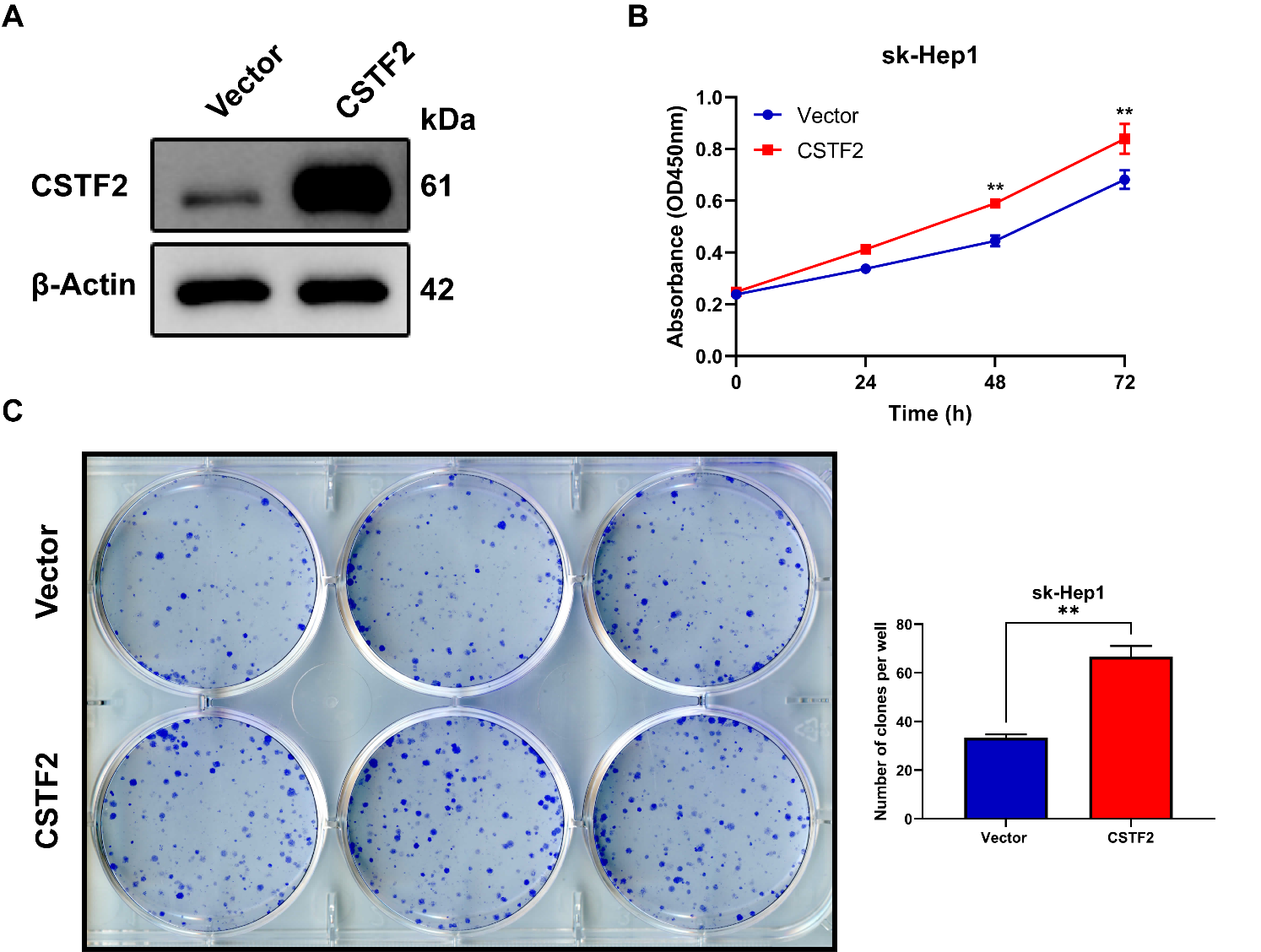


Figure S3. Overexpression of CSTF2 promoted the cell proliferation of HCC cells *in vitro*. (A) The stable overexpression of CSTF2 in sk-Hep1 cells was determined by western blot. (B) CCK-8 assay of the cell proliferation rate of overexpression of CSTF2 sk-Hep1 cells. (C) Colony-forming assay of overexpression of CSTF2 sk-Hep1 cells (left panel) and qualification of the number of colonies formed (right panel). CSTF2 represents overexpression of CSTF2. Empty vector was used as control. * $\text{p}$ < 0.05, ** $\text{p}$ < 0.01, ***$\text{ p}$ < 0.001 compared to Vector.
